# Disabled and Romani passengers face similar levels of discrimination but different levels of open hostility in the sharing economy

**DOI:** 10.1038/s41598-023-37263-1

**Published:** 2023-06-30

**Authors:** Borbála Simonovits, Benedek Kurdi, Gábor Simonovits

**Affiliations:** 1 Faculty of Education and Psychology, Eötvös Loránd University, Budapest, 1064 Hungary; 2 Department of Psychology, Yale University, New Haven, CT 06511 USA; 3 Department of Political Science, Central European University, 1100 Vienna, Austria; 4 Rajk College for Advanced Studies, Budapest, 1085 Hungary; 5 Centre for Social Sciences, Institute for Political Science, Budapest, 1097 Hungary

**Keywords:** Human behaviour, Psychology

## Abstract

This multimethod project investigates discrimination against members of two populous minority groups in the European Union: the Roma (numbering 6 million) and the disabled (numbering 100 million) on a leading Hungarian carpooling platform. In a field experiment, 1005 ride requests were sent to drivers, with passenger group membership (control, disabled, Roma) manipulated between participants. Widespread discrimination against both groups was apparent in significantly lower approval rates for disabled (56%) and Roma passengers (52%) relative to control (70%). Mechanisms driving anti-disabled and anti-Roma discrimination were probed using an experimental manipulation, natural language processing analysis of driver–passenger interactions, and an online survey (N = 398). Individuating information in the form of reviews did not mitigate unequal treatment, thus providing evidence against statistical (stereotype-based) discrimination. Militating against taste-based (attitudinal) discrimination, respondents reported negative attitudes toward Roma passengers but positive attitudes toward disabled passengers. Moreover, despite equivalent approval rates, disabled passengers were more likely to receive a response from drivers and received more polite responses than Roma passengers did. Overall, the observed patterns are most readily explained by intergroup emotions: Contempt toward Roma passengers likely engenders both passive and active harm, whereas pity toward disabled passengers likely engenders passive harm and active facilitation.

## Introduction

Over the past decade, businesses such as Airbnb, Uber, and WeWork have induced profound changes to the global economic order. Subsumed under the label of the sharing economy, these companies facilitate peer-to-peer provision of goods and services, coordinated through online platforms and communities^1^. Attesting to the importance of the sharing economy, it is projected to grow at a breakneck pace—from a value of $14 billion in 2014 to a value of $335 billion by 2025^2^, which is equivalent to the annual GDP of several mid-sized countries, such as South Africa and Hong Kong. Indeed, the sharing economy represents a radical departure from the conventional business model, with the emphasis shifting away from ownership and toward renting, bartering, and gifting^3^.

Some scholars have highlighted the positive nature of this paradigm shift and have pointed to the potential of the sharing economy to democratize socioeconomic relations^4–6^. Other authors have emphasized the problematic aspects of the sharing economy, including increasing economic inequality^7^, a lack of attention to sustainability^8^, and precarious labor conditions^9^. Most important from our perspective, the rapid growth of the sharing economy has outpaced attempts at regulation, including in the domain of group-based discrimination.

Since World War II, prohibitions against unequal treatment based on immutable characteristics—such as gender, ethnicity, or disability—have been a cornerstone of civil rights legislation in Western democracies. Following a long tradition of such legislation at the member state level, the European Union started adopting relevant regulations in the early 2000s, culminating in the 2012 EU Charter of Fundamental Rights, which prohibits group-based discrimination, including due to disability and ethnic origin. Parallel legislation also exists in the United States, in the form of the Civil Rights Act of 1964 and the Americans with Disabilities Act of 1990.

Although relevant regulations have been in place for decades, given their special legal status, businesses of the sharing economy have largely been able to sidestep antidiscrimination laws applicable to their more traditional counterparts (e.g., hotels, landlords, or taxicab companies)^10–12^. In fact, the sharing economy has been remarkably successful in escaping legal responsibility for harms incurred by its users, including instances of discrimination. Although public pressure has prompted some businesses to implement anti-discrimination policies^13, 14^, such policies remain relatively rare and tend to lack mechanisms for enforcement or evaluation.

As such, the sharing economy represents a unique opportunity for empirical research on group-based inequality and discrimination, for multiple reasons. First, from a theoretical perspective, the underregulated nature of the sharing economy allows for tests of whether antidiscrimination regulations applicable to more traditional economic actors have created sufficiently strong social norms to curb discrimination even in domains where they do not have binding legal power^15^.

Second, from a translational perspective, compiling solid evidence on the scope, strength, and antecedents of discriminatory behavior in the sharing economy may provide impetus for businesses to take steps against such discrimination and for regulators to consider solutions for applying existing antidiscrimination legislation to the sharing economy or to design specific regulations for this sector. Indeed, given that the sharing economy is supported by online platforms, interactions leave digital footprints, which allows researchers—and, in principle, market actors—to monitor discriminatory behavior. Such efforts are especially important in countries where government agencies cannot be relied on to enforce anti-discrimination legislation.

In line with these dual objectives, an emerging literature has started to document discrimination on different platforms of the sharing economy (for a recent review, see Ref.^16^). In their seminal work, Edelman et al.^17^ found that Airbnb users with stereotypically African American names were 16% less likely to have their requests for accommodation approved than otherwise identical users with stereotypically White American names (see also Ref.^14^). Underscoring the ubiquity of discrimination in the sharing economy, similar effects have been obtained in field experiments involving users with stereotypically African American names on Uber in Boston, MA^18^, African American and LGBTQ users on a rideshare platform in Washington, D.C.^19^, same-sex couples on Airbnb in Ireland^20^, users with Turkish names on a carpooling app in Germany^21^, and users with Chinese and Arabic names on a carpooling platform in Hungary^22^.

In the present paper, we expand on such work by conducting a multimethod investigation of discrimination against the Roma and the disabled in a country of the European Union. Specifically, in a randomized controlled trial over 1000 requests for rides were sent to drivers from fictitious profiles created for the purposes of the experiment on a popular Hungarian carpooling platform, with identity of the passenger (control, disabled, Roma) manipulated between participants. This design allows us to estimate the extent of anti-disabled and anti-Roma discrimination in a real-world context^23, 24^.

Moreover, via an additional manipulation embedded in the main experiment, text analysis of driver–passenger interactions, and a complementary survey administered to a separate sample drawn from the population of interest, we can start identifying the root causes of unequal treatment. Relying on this multimethod approach can foster a better understanding of fundamental societal and psychological processes giving rise to group-based inequality and can help formulate recommendations designed to alleviate discriminatory behavior in the sharing economy.

Disabled and Romani individuals have been selected as targets of the present project for several reasons. First, although the disabled and the Roma are among the most populous minority groups in the European Union (with the Roma numbering 6 million and the disabled numbering 100 million) and, indeed, the world, to date little evidence, and especially experimental evidence, on anti-Roma and anti-disabled discrimination in the sharing economy is available.

A previous experiment addressing anti-disabled discrimination in the sharing economy has documented widespread unequal treatment of individuals with blindness, cerebral palsy, dwarfism, and spinal cord injuries on Airbnb in the United States^13^. However, importantly, in this work hosts could have reasonably expected to incur substantial costs in making accommodations more accessible to guests with disabilities. In the present project, we eliminate this potential explanation for anti-disabled discrimination by implementing appropriate controls.

Specifically, as part of the experimental manipulation, drivers were informed that disabled passengers did not require any assistance getting into and out of the car. Moreover, the messages sent out in the control condition specified that the passenger was traveling with a large item, matched in size with the wheelchair mentioned in the disabled condition. As such, any differences between the control and disabled conditions are unlikely to be due to perceived differences in the levels of inconvenience associated with traveling with each passenger.

Second, beyond the sheer size of these two groups, investigating discrimination against the Roma and the disabled in the domain of physical mobility is of special importance given that these two groups are disproportionately likely to face mobility issues stemming from low levels of car ownership^25^. Such mobility issues, in turn, can create cascades of disadvantage encompassing multiple domains of social and economic life, including education, employment, and healthcare^26, 27^.

Third, and critically, including multiple targets in examinations of discriminatory behavior can help us move beyond mere demonstrations of group-based disparity and toward understanding its root causes. As pointed out by Bertrand and Duflo^28^, investigations of discrimination in field settings rarely attempt to address the potential mechanisms giving rise to such discrimination (for notable exceptions, see Refs.^29, 30^). Such lack of attention to mechanism is unfortunate both because understanding the psychological underpinnings of discriminatory behavior is of inherent theoretical interest and because successful interventions against such behavior require an understanding of the processes from which it emerges. As such, in the present work we go beyond simply probing for the presence of unequal treatment of disabled and Romani targets and collect multiple forms of evidence—an experimental manipulation, text analysis of passenger–driver interactions, and survey responses—on why discrimination might occur.

Unequal treatment of disabled and Romani individuals may be a manifestation of taste-based discrimination, i.e., it may emerge from negative intergroup attitudes^31^. This notion is in line with standard social psychological models positing that attitudes are a major driver of intergroup behavior^32^ as well as meta-analytic evidence from hundreds of studies establishing a link between negative intergroup attitudes and discrimination^33^. Indeed, negativity toward the Roma across countries of the European Union is well-documented—so much so that anti-Roma bias can be described as normative, whereas egalitarian views are counter-normative^34^. By contrast, attitudes toward the disabled tend to be positive, or at least considerably less negative^35^. As such, the taste-based discrimination perspective predicts that drivers should discriminate against Romani passengers but not against disabled passengers.

By contrast, statistical discrimination refers to the idea that discrimination need not stem from animus but rather may emerge from the lack of relevant information^36, 37^. Specifically, in the present study, drivers may believe that, on average, disabled and Romani individuals are less desirable as passengers than their non-disabled and non-Romani counterparts. However, once they receive pertinent information on the specific interaction partner, this information rather than the stereotype will guide social judgement and behavior. This perspective, too, is in line with standard social psychological approaches^38^. Critically, according to the statistical discrimination perspective, discrimination should be eliminated, or at least alleviated, when passenger ratings are made available to drivers. This idea has received some empirical support in past work on the sharing economy^14, 39, 40^ and, as such, we included it as a further test of mechanism (and as a potential intervention) in the present work.

For example, in work that has directly informed the design of the present studies, Tjaden et al.^29^ have demonstrated widespread discrimination against drivers with Turkish and Iranian names. Moreover, in line with the statistical discrimination perspective, these authors have found that the presence of positive ratings on a driver’s profile mitigated the amount of discrimination experienced by them. In fact, at the highest levels of ratings, the treatment of native vs. ethnically Turkish or Iranian drivers was indistinguishable. Importantly, however, the work by Tjaden et al. was an observational study relying on a preexisting dataset of already completed rides and is therefore subject to the risk of omitted third variables. Moreover, whereas Tjaden et al. examined discrimination against drivers along a single dimension of ethnicity, the present work focuses on discrimination against passengers on the basis of multiple stigmatized identities.

Finally, although some work has criticized exclusive reliance on these two theoretical ideas (taste-based and statistical discrimination) in field experiments on discrimination^28, 41^, so far a clear third option has yet to emerge. Here we focus on a set of approaches from social cognition emphasizing the importance of group-based emotions to understanding and predicting patterns of discrimination. Within this group of theories, the one that seems most directly applicable to the present case is the BIAS map^42, 43^. This theory, which to our knowledge has yet to be tested in real-world contexts, makes unique predictions for the patterns of real-world discrimination that should emerge under the conditions of the present work.

The BIAS map conceptualizes social group stereotypes along two major dimensions: warmth and competence. Warmth stereotypes are thought to guide active behavioral tendencies, with warm groups eliciting active facilitation and cold groups eliciting active harm. Competence stereotypes, in contrast, are thought to guide passive behavioral tendencies, with competent groups eliciting passive facilitation and incompetent groups eliciting passive harm. Given that the Roma are stereotyped as both cold and incompetent and the disabled are stereotyped as warm but incompetent^44^, different patterns of discrimination are expected to emerge toward the two groups.

Specifically, the Roma should engender the intergroup emotion of contempt, along with both active and passive harm in intergroup behavior, whereas the disabled should engender the intergroup emotion of pity, along with active facilitation but passive harm in intergroup behavior. In the context of the present studies, these ideas translate into predictions of similar levels of requests for rides being denied (a form of passive harm) but different levels of open hostility both in driver–passenger interactions and expressions of group attitudes on the survey measure (forms of active harm). Moreover, based on the BIAS map, we anticipate higher levels of contempt to be reported toward Roma targets and higher levels of pity to be reported toward disabled targets on a questionnaire measure.

To summarize, the present project aims to investigate discrimination against members of two of the largest minority groups in the European Union—disabled and Romani individuals—in the sharing economy, specifically on a popular Hungarian carpooling platform. These two minority groups are protected by EU antidiscrimination policies and are nonetheless especially severely affected by issues related to limited physical mobility, which in turn has wide-ranging consequences for their participation in virtually all aspects of social and economic life.

Beyond documenting the presence of discrimination, we pursue a multipronged approach, combining theories from economics and social cognition as well as methods ranging from field experimentation and natural language processing to questionnaires and survey experiments to foster a mechanistic understanding of unequal treatment. Based on the insights derived from our investigation, we formulate theoretical lessons for understanding basic phenomena of group-based disparity and consider potential solutions to help mitigate discrimination in the sharing economy.

## Results

### Field experiment

**Figure 1:**
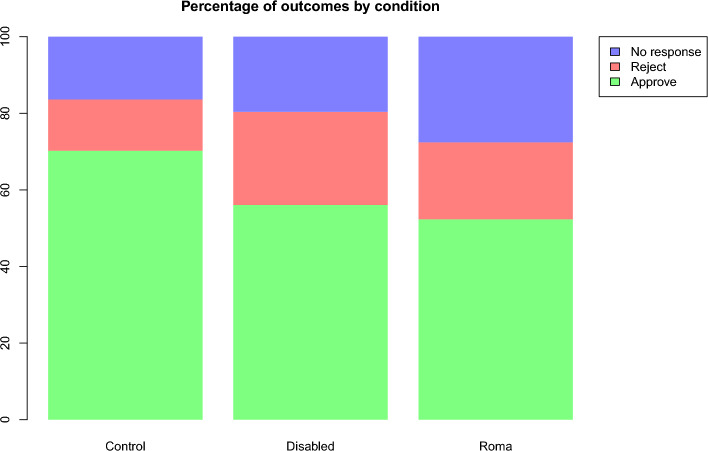
Percentage of outcomes (approval, rejection, no response) by passenger group membership (control, disabled, Roma) in the field experiment (total N = 1005 requests).

In the field experiment, we obtained clear and robust evidence for discrimination against disabled and Romani targets (see Fig. 1). Specifically, whereas requests from control individuals were accepted 70.24% of the time, 95% confidence interval (CI) [65.13%; 74.89%], acceptance rates dropped considerably in the two remaining conditions, to 56.07% [50.59%; 61.41%] for disabled passengers and to 52.30% [47.05%; 57.50%] for Romani passengers. Overall, these three conditions significantly differed from each other, χ^2^(1) = 25.62, p < 0.001. Whereas the pairwise comparisons between the control and disabled conditions, z = 3.75, p < 0.001, and the control and Roma conditions, z = 4.78, p < 0.001, were statistically significant, the disabled and Roma conditions did not significantly differ from each other, z = 0.98, p = 0.328, suggesting similar levels of discrimination against these two groups.

In Supplementary Information, we report robustness checks of this finding. To summarize, the effect was robust to stimulus effects (the specific names and images used to represent each target), as well as to a wide array of driver characteristics (including sex, rating, and experience) and trip characteristics (including trip length, price, and the number of open seats available in the car). Although some of these variables had main effects, the effect of condition remained statistically significant in each case, and none of these variables moderated the effect of condition on acceptance rates, attesting to the ubiquity of discrimination based on disability and ethnic origin.

As an initial test of mechanism and as a potential intervention, we probed whether the presence of positive user reviews included in targets’ profiles would alleviate discrimination relative to a control condition in which no user ratings were provided. This manipulation did not moderate the effect of target group membership (control, disabled, Roma), with the best-fitting model including only a main effect of the target group variable but no interaction, χ^2^(1) = 3.17, p = 0.366. This result is incompatible with the statistical discrimination perspective under which individual-level information should have reduced, or perhaps even eliminated, group-based disparities.

Although Romani and disabled passengers were treated similarly in terms of acceptance rates, large differences emerged when it came to receiving any response to their message on the platform (irrespective of whether the request was approved or not; see Fig. 1), χ^2^(1) = 13.43, p = 0.001. Specifically, control passengers received a response 83.63% [79.28%; 87.21%] of the time, disabled passengers 80.37% [75.66%; 84.36%] of the time, and Romani passengers 72.41% [67.48%; 76.85%] of the time. In this case, the difference between the control and disabled conditions was not significant, z = 1.09, p = 0.278, whereas the difference between the control and Roma, z = 3.50, p < 0.001, and Roma and disabled conditions was, z = 2.41, p = 0.016. Like for acceptance rates, the effect was not moderated by whether individual-level information was provided or not, χ^2^(1) = 2.17, p = 0.536.

The fact that disabled and Romani passengers received responses to their ride requests at different rates is indicative of a difference in how discrimination against the two groups manifests itself: Higher response rates for disabled compared to Romani passengers demonstrate that drivers express lower levels of open hostility toward the former than to the latter group while inflicting similar levels of passive harm to both by declining requests for rides. As a further test of this idea, we compared the length and content of the messages sent by drivers to passengers across the three different conditions. The politeness of the response was assessed both in an automated manner using natural language processing (NLP) methods and manually by human coders.

**Figure 2:**
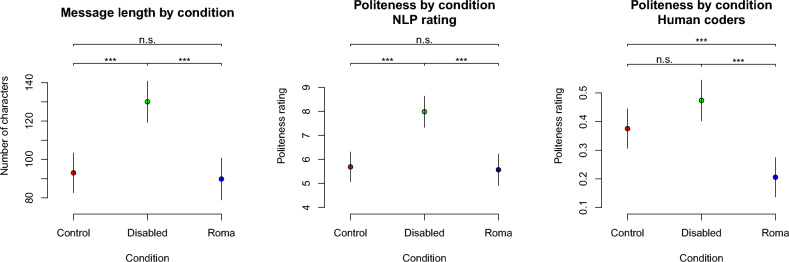
Secondary dependent variables from the field experiment (total N = 1005 requests) by condition, including message length (left pane), NLP-assessed message politeness (middle pane), and human-coded message politeness (right pane).

Message length significantly differed by passenger group membership (see Fig. 2), χ^2^(1) = 32.72, p < 0.001. Specifically, disabled passengers tended to receive the longest messages (n_char_ = 130 [119; 141]), followed by control passengers (n_char_ = 93 [83; 103]), and finally by Romani passengers (n_char_ = 90 [79; 101]). The disabled condition significantly differed from each other condition (ps < 0.001), whereas the difference between the control and Roma conditions was not significant (p = 0.676). This result suggests that drivers tended to go out of their way to provide lengthy responses to disabled passengers to a degree that they did not for Romani or control passengers.

The results of the politeness ratings reinforced the same conclusion (see Fig. 2). Specifically, we obtained an overall condition difference on the machine-coded politeness measure, χ^2^(1) = 33.22, p < 0.001. On this measure, disabled passengers received significantly more polite responses than control, t(788) = 4.99, p < 0.001, or Roma passengers did, t(788) = 5.11, p < 0.001. The difference between the control and Roma conditions was in the expected direction but did not reach significance, t(788) = 0.25, p = 0.797.

A condition difference also emerged on the human-coded politeness measure, χ^2^(1) = 28.55, p < 0.001. Disabled passengers received more polite responses than did control passengers, although this difference did not reach statistical significance, t(887) = 1.94, p = 0.052. Romani passengers received significantly less polite responses than did control passengers, t(887) = 3.41, p < 0.001, or disabled passengers, t(887) = 5.29, p < 0.001. Critically, both measures converged on the result that disabled passengers received more polite responses than Romani passengers did, thus further underscoring the difference between the two groups in the extent of active harm experienced.

### Survey study

As a further test of mechanism, we collected self-reported attitude and intergroup emotion ratings from a separate set of participants (N = 398) drawn from a population of Hungarian adults who own and regularly drive cars and are therefore potential users of the carpooling app. In line with local norms against the sharing of data on membership in protected classes, no information on ethnicity or disability status was collected. However, based on car ownership data^25^, it is safe to assume that most participants were ethnically Hungarian and non-disabled. Moreover, the inclusion of some Roma or disabled participants in the sample could have resulted, if anything, only in more conservative estimates of the effects reported below.

**Figure 3:**
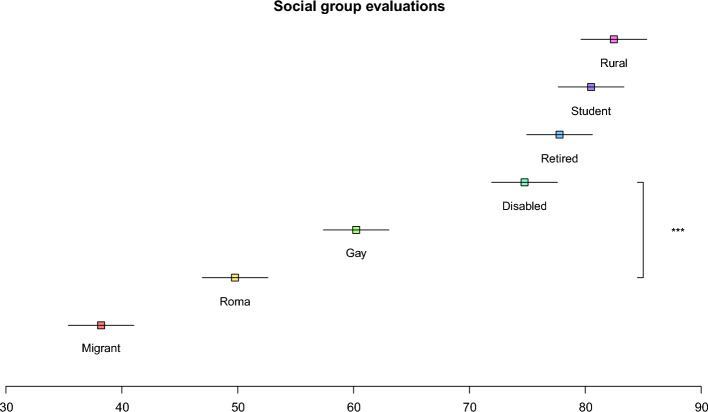
Attitudes toward people from rural areas, students, retired people, disabled people, gay people, Romani people, and migrants in the survey study (N = 398). Evaluations are displayed on a 100-point scale, with higher scores corresponding to more positive evaluations. Error bars show 95-percent confidence intervals. To create maximal correspondence with the survey experiment, group-based attitudes were measured as willingness to share a ride with a person from a specific group.

Attitude ratings are displayed in Fig. 3. Critically, participants expressed significantly more positive attitudes toward the disabled (M = 74.8 [71.9; 77.6]) than they did toward the Roma (M = 49.8 [46.9; 52.6]), t(2383) = 16.43, p < 0.001. In fact, evaluations of the disabled were similar to societal reference groups (such as students and retirees), whereas evaluations of the Roma were highly unfavorable, second only to migrants in levels of negativity. This result is reminiscent of classic findings of attitude–behavior dissociation^45^ and makes the data from the field experiment difficult to reconcile with a taste-based discrimination account. After all, if discrimination is driven by attitudinal negativity, then Romani individuals should have encountered more widespread discrimination than disabled individuals did. Contrary to this idea, we found equivalent levels of discrimination despite vastly different attitudes toward the two groups.

**Figure 4:**
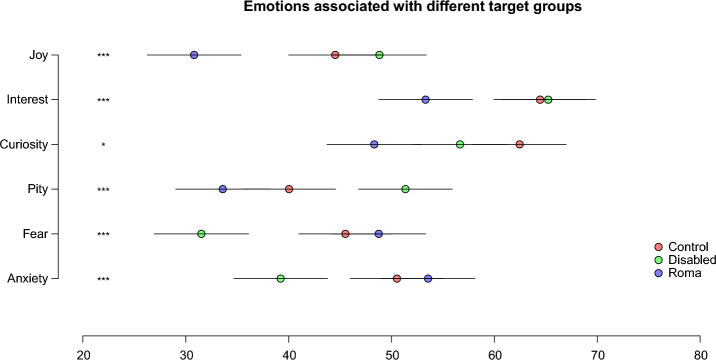
Intergroup emotions expressed toward control, disabled, and Roma targets in the survey study (N = 398). Endorsement of different emotions is displayed on a 100-point scale, with higher scores corresponding to higher levels of each emotion. Error bars show 95-percent confidence intervals. Significant differences between disabled and Roma targets are marked ***(p < 0.001) and *(p < 0.05), respectively.

The two groups also differed from each other in all seven of the intergroup emotions tested (see Fig. 4). Specifically, disabled targets were rated higher than Romani targets on all approach-oriented, positive emotions (joy, interest, and curiosity) and lower than Romani targets on both avoidance-oriented, negative emotions (fear and anxiety). Critically from the perspective of the BIAS map^42, 43^, the disabled were rated considerably higher than the Roma on pity—an ambivalent emotion precited to give rise to a mix of active facilitation and passive harm in intergroup behavior. Overall, intergroup emotions toward the Roma were found to be unequivocally negative, whereas intergroup emotions toward the disabled were found to be mixed, indicative of ambivalence.

This pattern of results is remarkably well aligned with the behavioral findings obtained in the field experiment in which Romani passengers were subject to blatant discrimination encompassing both active and passive harm, including higher rates of denied requests, lower probability of receiving a response, and both shorter and less polite responses even when a response was received. By contrast, patterns of discrimination against disabled targets were mixed. Although the level of passive harm in the form of denied requests was the same as for Romani passengers, such passive harm was accompanied by active facilitation, including a higher probability of receiving a response, along with longer and more polite responses than not only in the Roma condition but even in the control condition.

## Discussion

A field experiment conducted on a popular Hungarian carpooling platform has provided evidence for widespread discrimination against passengers with disabilities and Romani passengers: Whereas control passengers’ requests for rides were approved 70% of the time, the same ratio dropped significantly and considerably to 52% for Roma and to 56% for disabled passengers. This finding generalized across several model specifications as well as driver and ride characteristics, underscoring the robustness and pervasiveness of group-based disparities.

Group-based discrimination has pernicious consequences for the targets of bias. First, discrimination has adverse psychological effects, including the fact that it creates attributional ambiguity^46^. That is, when members of stigmatized groups, such as disabled and Romani individuals, encounter negative treatment, such as denial of a ride request on a carpooling app, they face uncertainty regarding the cause for such negative treatment, specifically whether it was their stigmatized identity or some other, unrelated, reason. This uncertainty, in turn, can produce additional adverse effects, including anxiety and rumination.

Second, the targets of bias incur opportunity costs as a result of the discrimination to which they are subjected. Specifically, based on the data obtained in the field experiment, non-disabled, non-Roma individuals can expect to send out only 1.38 requests for rides before a request is approved; for disabled individuals, the same number is 1.76 and for Roma individuals, 1.90. Although this difference may seem minor, multiple disadvantages can compound across different areas of a stigmatized person’s life and create massive disparities in the aggregate^47^.

Third, disabled and Romani individuals are particularly likely to face challenges related to physical mobility due to lower levels of car ownership^25^. Such lack of mobility, in turn, creates additional disadvantages across all areas of daily life, including employment, education, and healthcare^26, 27^. As such, it is especially pernicious for members of these two groups to face exclusion from sharing economy platforms that could facilitate their physical mobility without requiring car ownership.

Finally, discrimination of the kind documented here, consisting in denying members of stigmatized groups the opportunity to have—potentially positive—interactions with members of majority groups can create a vicious circle of mistrust. Positive interactions between two individuals of equal status sharing a common goal have been shown to decrease intergroup bias^48^. However, if members of dominant groups refuse to engage in these interactions in the first place, this path toward improved intergroup relations is blocked.

The present findings corroborate existing work providing evidence for discrimination in the sharing economy^13, 14, 17–22^. At the same time, we also go beyond such work by exploring discrimination against target groups that have rarely been investigated and, critically, by probing not only the presence and magnitude but also the root causes of unequal treatment. Overall, the observed patterns are difficult to reconcile with both standard theoretical perspectives in the economics literature on discrimination: taste-based (attitudinal) and statistical (stereotype-based) discrimination.

Specifically, the taste-based model of discrimination predicts that if two social groups face similar levels of discrimination, they should be disliked to similar degrees and vice versa. Contrary to this prediction, we found equivalent levels of discrimination against the disabled and the Roma although participants expressed open hostility toward the latter but not at all toward the former. The results are similarly difficult to reconcile with the statistical discrimination model given that the levels of discrimination were equivalent irrespective of whether drivers received positive individual-level information about passengers or not. Of course, a different or stronger manipulation of individuating information may have produced different effects; as such, we hope that future work will further explore relevant effects.

Nonetheless, the present findings were most directly compatible with theories of intergroup emotion, specifically the BIAS map^42, 43^, which posits that discrimination emerges not from mere attitudinal negativity or a lack of individuating information but rather from the emotions evoked by different social groups. In the survey, participants expressed all-encompassing negativity toward Romani targets, placing this group in the contempt quadrant of the BIAS map. In contrast, disabled targets were subject to pity—an ambivalent emotion with mixed behavioral consequences.

Accordingly, Romani passengers faced ubiquitous discrimination on the carpooling app, including higher rejection rates, lower response rates, and shorter and less polite messages. Meanwhile, although disabled passengers faced similar levels of passive harm to Romani passengers in the form of equivalent rates of rejected ride requests, they were rarely subjected to open expressions of hostility. In fact, disabled passengers were more likely to receive messages not only than Roma passengers but even than non-disabled control passengers; and messages were longer and more polite than messages received by members of either other group.

The relevant economics literature often assumes that taste-based and statistical forms of discrimination are both exhaustive and mutually exclusive (but see Refs.^28, 49^). The present data caution against the former assumption given that we obtained a pattern of results that was not easy to reconcile with either model. In addition, research in social cognition has provided ample evidence for a robust relationship between attitudes and stereotypes^50^, which also makes it hard if not impossible to empirically separate taste-based and statistical discrimination from each other: In the present study, drivers may have believed that disabled and Romani passengers are undesirable interaction partners because of attitudinal negativity toward the two groups rather than due to any specific stereotype.

More generally, field experiments on discrimination may benefit from the inclusion of additional theoretical and empirical perspectives from social cognition research not directly considered here. For example, attitudinal differences between the disabled and the Roma observed in the present work may have stemmed from differential degrees of motivation to respond without prejudice to the two groups^51^, which may well be an indication of differences in social norms condoning discrimination against them^34, 35^. If this is the case, implicit measures of attitudes and stereotypes, which are known to help circumvent social desirability concerns as well as strategic responding on the basis of perceived social norms, may be used to provide additional insights into the root causes of discriminatory behavior^52, 53^.

Alternatively, or in addition, social norms may have mediated the effects of intergroup emotions of discriminatory behavior—a possibility that we believe may be fruitfully explored in future work. Specifically, it seems conceivable that societal norms against bias and discrimination may differ in strength (or even direction) depending on a social group’s placement on the BIAS map: Whereas relatively strong norms against open hostility might exist for social groups subject to ambivalent social group emotions (and especially pity), the same norms are likely to be weaker or even nonexistent for social groups subject to contempt.

Finally, despite some evidence for successful applications in past work^14, 39, 40^, providing passenger ratings to drivers did not reduce discrimination to any appreciable degree. We can only speculate about the reasons for this discrepancy, but motivational factors likely played a role. Specifically, if drivers are not motivated to control their biased behavior^54^, they may simply disregard the individuating information provided to them. Reviews may also be less effective in combating discrimination than previously assumed for an additional reason: If the same factors that create discriminatory behavior also give rise to biased reviews, then, if anything, reviews may exacerbate group-based disparities.

As such, multiple interventions may be considered to curb discriminatory behaviors of the kind observed here. First, platforms may choose to implement debiasing programs designed to change affect and other group-based cognitions underlying discriminatory behavior. However, the effects of such interventions are often short-lived^55^; moreover, the present data suggest that eliminating attitudinal negativity may not be sufficient to produce the desired behavioral change. Instead, the complex nature of mixed intergroup emotions, such as pity or envy, may have to be considered.

Second, sharing economy platforms may opt for blinding users to each other’s social group memberships. After all, if drivers are unaware of passengers’ stigmatized identities, then they cannot discriminate against them based on those identities^18, 56^. However, trust is an indispensable element of transactions in the sharing economy^57, 58^. Therefore, omitting information about group-based identity may have to be combined with steps to furnish information that can signal the trustworthiness of interaction partners. Such information can include objective measures (such as number of previous transactions completed, punctuality, or responsiveness to messages), which may be less severely affected by group-based biases than inherently subjective reviews and ratings.

## Methods

### Institutional approval and informed consent

The project received ethical approval from the Ethical Research Committee at Eotvos Lorand University, and all research was performed in accordance with relevant guidelines and regulations. As is standard practice in audit studies^59^ and in line with the wishes of the carpooling platform to protect its reputation, participants in the field experiment did not provide informed consent, nor were they debriefed about the purpose of the study. Participants in the survey study provided informed consent. All research activities were performed with the knowledge and approval of the carpooling platform.

### Open science practices

All materials, data, and analysis code are available from the Open Science Framework (https://osf.io/m7hkj/). The design of the field experiment, including the statistical analyses involving the passenger identity and passenger review variables, were preregistered (https://osf.io/5upvz/). The analyses involving message length and politeness and the analyses of the survey study were not preregistered.

### Field experiment

The field experiment (N = 1005) relied on a 3 (passenger identity: control, disabled, Roma) × 2 (passenger review: positive vs. absent) between-participant design.

The fictitious rider profiles created for the purposes of the experiment contained a facial image, a name, and, in the relevant condition, reviews purportedly written following past rides (for more details, see Supplementary Information). These profiles were then used to send out short messages to drivers, consisting of a greeting and details of the trip (e.g., “Hello, I would like to travel from X to Y”).

In the Roma condition, two cues to passenger ethnicity were provided. First, in preparing passenger profiles, a graphic designer created Roma and non-Roma versions of a set of facial images obtained from male volunteers. In an online pretest, we verified that the Roma versions of the faces were, in fact, more likely to be perceived as Roma and the non-Roma versions more likely to be perceived as ethnically Hungarian. As an additional manipulation of ethnicity, we used stereotypically ethnically Hungarian and stereotypically ethnically Romani first and last names.

In the disabled condition, we used the same facial images and names as in the control condition. In this condition, the key manipulation was embedded in the message sent to drivers. Specifically, the request mentioned that the passenger would arrive in a wheelchair, which would have to be transported in the trunk of the car. The message made it clear that the passenger would not require any assistance from the driver. In addition, critically, messages in the control condition mentioned that the passenger would be traveling with a large item, similar in size to a wheelchair. As such, differences between the control and disabled conditions are unlikely to be due to the real or perceived inconvenience associated with transporting the wheelchair.

Finally, we manipulated passenger reviews in the following manner. Similar to other online marketplaces, profiles on the carpooling platform via which the experiment was conducted can contain both numerical ratings and text-based reviews written following past rides. Half of the profiles created for the purpose of the experiment included four positive reviews (e.g., “good company,” “pleasant journey”) and one neutral review (e.g., “he postponed the trip, but he cancelled in time”), purportedly written by past interaction partners as well as a numeric rating of 4.5 out of 5. The other half of the profiles contained no reviews or ratings.

The field experiment was conducted by Szinapszis, a Hungarian market research firm, between October 12 and November 6, 2021. They took samples from the population of available rides on the platform and contacted drivers according to a pre-determined schedule that included a list of rides to be completed by each test passenger profile. We excluded commercial rides, international rides, and rides within a single town. Members of the research team conducting the study were instructed to carry on communication with the driver until its outcome could be determined (ride approved or rejected), and then politely cancel the trip. The outcome of interest was coded as “approved” if the driver agreed to give a ride to the passenger and as “rejected” otherwise.

We also recorded characteristics of the driver (sex, average rating, and experience), characteristics of the trip (length, price, number of open trips available), as well as the text of any messages exchanged between drivers and passengers. The text of the messages sent by drivers was manually coded for politeness by four independent coders blind to condition (interrater reliability: Kendall’s W = 0.593), and the mean ratings provided by the coders used in the relevant analyses. Automated politeness ratings were obtained using the politeness package^60^ in the R statistical computing environment after automatically translating the text of the messages from Hungarian into English using the corresponding Google Translate API.

### Survey

To measure group-based attitudes and emotions toward the two target groups of interest (the disabled and the Roma) and to benchmark these attitudes and emotions against other social groups, an online survey was administered to a convenience sample of Hungarian adults (final N = 398). The data were collected in December 2021 by Szinapszis, a Hungarian market research firm, as part of a larger experiment with multiple conditions. Given our goal to approximate discriminatory behavior among potential drivers on the carpooling app, data were collected from a diverse sample of respondents that own and use a car.

After providing informed consent and passing an attention check, participants were asked to watch a short movie clip about the carpooling platform, which did not make any reference to stigmatized groups or discrimination. Of central theoretical interest were responses to two sets of survey items. Information on the remaining items included in the survey is provided in Supplementary Information.

First, we asked participants about their willingness to share a ride with members of seven social groups, including not only the disabled and the Roma, but also people from rural areas, students, retired people, gay people, and migrants. This item served as a measure of general group-based attitudes, indexed in a way as to create maximal correspondence between the survey and the field experiment. Second, an experiment was embedded within the survey in which we asked participants how carpooling with an individual would make them feel. They were assigned to answer this question about either “a person,” “a person with a disability,” or a “Romani person.” We measured six emotional responses (joy, interest, curiosity, pity, fear, and anxiety) to this hypothetical scenario^13^.

## Supplementary Information

Supplementary Information.

## Data Availability

All data are available from the Open Science Framework (https://osf.io/m7hkj/).
