# Supplementary material for: Disabled and Romani passengers face similar levels of discrimination but different levels of open hostility in the sharing economy
Source: Sci Rep. 2023 Jun 30;13:10605. doi: 10.1038/s41598-023-37263-1 (PMC10313893; doi:10.1038/s41598-023-37263-1)
Supplement: Supplementary file 1 — Supplementary Information. [file 41598_2023_37263_MOESM1_ESM.docx]

**Supplementary Information for**

Disabled and Romani passengers face similar levels discrimination but different levels of open hostility in the sharing economy

Bori Simonovits*, Benedek Kurdi, and Gábor Simonovits

*Email: [simonovits.borbala@ppk.elte.hu](mailto:simonovits.borbala@ppk.elte.hu)

Field Experiment

**Supplementary Methods.** In the control and disabled conditions, one of four passenger names (Bence Kovács, Péter Molnár, Ákos Nagy, and Máté Varga) was selected randomly. These names had been chosen to suggest that the passenger is ethnically Hungarian. In the Roma condition, one of four passenger names was selected randomly from a different set (Kevin Gáspár, Richárd Kolompár, Szebasztián Oláh, and Márió Orsós). These names had been chosen to suggest that the passenger is ethnically Roma.

As a further manipulation of ethnicity, two versions (Roma vs. non-Roma) of four faces were created. The Roma versions of the faces were used in the Roma condition and the non-Roma versions of the faces were used in the control and disabled conditions. In a pretest, the Roma versions of the four faces were found to be considerably more likely to be rated as Roma (means = [75.47; 75.89; 66.38; 76.87], SDs = [25.42; 24.86; 27.57; 21.64]) than the non-Roma versions (means = [21.00; 28.34; 30.05; 43.71], SDs = [23.03; 25.22; 23.29; 23.51]). Each pairwise difference was statistically significant (ps < 0.001) and large (Cohen’s ds ≥ 1.43). Due to a clerical error, only two of the four face pairs were used in the experiment. All four face image pairs are available from OSF (<https://osf.io/m7hkj/>).

In the control and Roma conditions, one of the following messages was sent to the driver (translated from the original Hungarian): “Hi! I am going to visit my family and I have a few things that I would like to bring with me. I have a large suitcase that should fit into the trunk. Would that work for you? Best, [Name]” or “Hi! I’m going on a longer hike, and I will have a hiking backpack with me. It should fit easily into the trunk. Would you be able to take me? Best, [Name].” In the disabled condition, the following message was sent to the driver: “Hi! I am disabled and therefore I need to take my wheelchair on the trip. When it’s folded up, the size is the same as a larger suitcase. It should fit into the trunk. I don’t need help to get into and out of the car. Would that be OK with you? Best, [Name].”

In addition to passenger group membership, the presence of individuating information in the form of reviews and ratings was manipulated orthogonally. In the no individuating information condition, no reviews or ratings were provided. In the condition with individuating information, the passenger’s numerical rating was set to 4.5 out of 5, and four positive and one neutral text-based reviews were added to the profile. These values were selected to maintain believability. As we note in the main text, even higher ratings or a larger number of reviews may have produced different effects — a possibility that we hope will be explored in future work.

**Supplementary Results.** The effect of group membership (control, disabled, Roma) remained significant after accounting for stimulus effects and several driver and ride characteristics.

Specifically, in a model including random slopes for target facial identity and target name, the effect of condition was still statistically significant, χ^2^(2) = 6.21, p = 0.044. The difference between the control and disabled conditions, z = 3.40, p < 0.001, and the control and Roma conditions, z = 2.88, p = 0.004, was significant, whereas the disabled and Roma conditions did not differ from each other, z = 0.71, p = 0.478. Moreover, the numerical estimates of approval rates were highly similar to those reported in the main text.

In addition, the results were robust to driver characteristics, including driver sex, driver rating, and driver experience.

The best-fitting model for driver sex included only an effect of condition and no main effect of sex and no interaction.

The best-fitting model for driver rating included a main effect of condition and a main effect of driver rating such that more highly rated drivers were more likely to accept requests (z = 4.05, p < 0.001). Critically, the rating variable did not interact with the condition variable and the patterns involving the condition variable remained the same as in all other models.

The best-fitting model for driver experience included a main effect of condition and a main effect of driver experience such that more experienced drivers were more likely to accept requests (z = 4.64, p < 0.001). Critically, the experience variable did not interact with the condition variable and the patterns involving the condition variable remained the same as in all other models.

The results were also robust to ride characteristics, including length of trip, price, and number of open seats.

The best-fitting model for length of trip included a main effect of condition and a main effect of length of trip such that requests for longer trips were more likely to be accepted (z = 7.67, p < 0.001). Critically, the trip length variable did not interact with the condition variable and the patterns involving the condition variable remained the same as in all other models.

The best-fitting model for price of trip included a main effect of condition and a main effect of price of trip such that requests for more expensive trips were more likely to be accepted (z = 8.51, p < 0.001). Critically, the price variable did not interact with the condition variable and the patterns involving the condition variable remained the same as in all other models.

The best-fitting model for number of open seats included a main effect of condition and a main effect of number of open seats such that requests with more open seats in the car were less likely to be accepted (z = -2.28, p = 0.023). Critically, the number of open seats variable did not interact with the condition variable and the patterns involving the condition variable remained the same as in all other models.

**Online Survey**

**Supplementary Methods.** In the online survey, participants first provided informed consent, then agreed to read and respond carefully to the questions and completed an attention check. They were then asked whether they had a car that they drove regularly to determine eligibility in the study.

Eligible participants were then asked general questions about carpooling applications, including whether they have used a carpooling app (as a passenger, as a driver, as both, or not at all), and whether they knew someone who had used a carpooling app as a passenger and/or as a driver. These questions served as buffers.

Then participants were asked to watch a short clip of about 45 seconds containing general information about the carpooling app. Two other groups of participants whose data are not included in this paper watched other clips that addressed discrimination based on disability and ethnicity, which served as experimental manipulations designed to shift responding on the dependent variables described below.

Following the clip, participants reported their overall liking of the clip on a 4-point scale and reported, also on 4-point scales, to what extent they found the clip funny, boring, sad, shocking, and exciting.

On the following screen, participants reported their demographics, including their sex, residence, year of birth, occupation, and highest educational attainment.

Participants then reported whether they knew anyone belonging to the following social categories: retired, Roma, disabled, student, rural, and gay.

Participants then used 4-point scales to respond to general items about carpooling services, including carpooling presenting a good opportunity for drivers to (*a*) meet people, (*b*) help protect the environment, and (*c*) save money, and (*d*) carpooling presenting unnecessary risks for drivers. Participants were then asked to select from a list services of the sharing economy that they had used themselves, including Airbnb, Oszkár, Vatera, Facebook Marketplace, MOL Bubi, and Greengo. Participants were then asked to rate how important each of the following criteria was to them when making decisions about what means of transportation to use: price, comfort, fun, and environmentalism (4-point scales). These items also served as buffers.

Participants were then presented with a hypothetical scenario in which they were asked to select up to 3 of 4 passengers to share a ride with on a carpooling app. Three of the passengers were ethnically Hungarian and one passenger was ethnically Roma. After they had entered their response, participants were asked to describe their reasoning briefly. In this paper we do not focus on this item given that it included both ethnicity and social class information — a variable outside of the scope of the present study.

Then, critically, participants were presented with another hypothetical scenario in which they were asked to indicate, on 4-point scales, to what extent they would feel each of the following emotions if they, as a driver, had to share a ride with a person: curiosity, anxiety, interest, fear, joy, and pity. In a between-participant manipulation, the person was described either as “a passenger” (control condition), “a disabled passenger” (disabled condition), or “a Romani passenger” (Roma condition). These intergroup emotion variables are discussed in detail in the main text.

Subsequently, participants were asked to indicate, using 100-point scales, how much they would like to share a ride with passengers from the following social groups: retired, Roma, gay, migrant, disabled, rural, and student. This intergroup attitude variable is also discussed in detail in the main text.

Finally, participants indicated, on a 4-point scale, to what extent it was important for carpooling platforms to ensure equal treatment of the same seven groups mentioned above. This variable was found to be redundant with the intergroup attitude variable; therefore, we do not discuss it in the main text.

All variables not discussed in the main text are available for download and reanalysis in the open data (<https://osf.io/m7hkj/>).
